# Comparative Effects of Freeze-Drying and Sun-Drying on Phenolic Composition, Antioxidant Capacity, Microbial Characteristics, and Aroma Profile of Purple Sweet Potato-Enriched Tarhana

**DOI:** 10.3390/foods15122217

**Published:** 2026-06-19

**Authors:** Eda Elgin Kiliç, Songül Kesen

**Affiliations:** Naci Topcuoglu Vocational School, Gaziantep University, Gaziantep 27600, Türkiye

**Keywords:** tarhana, freeze-drying, purple sweet potato, volatile compounds, antioxidant capacity, phenolic compounds, lactic acid bacteria

## Abstract

This study investigated the effects of drying method and ingredient form on the quality characteristics of tarhana enriched with purple sweet potato (*Ipomoea batatas* L.). Tarhana samples were formulated with purple sweet potato in two forms (puree and freeze-dried powder) at incorporation levels of 5% and 10%, and subjected to either traditional sun-drying or freeze-drying. The drying method emerged as the dominant factor influencing product quality. Freeze-dried samples exhibited significantly lower moisture content and water activity along with a highly porous microstructure, indicating favorable physicochemical characteristics associated with product stability. Purple sweet potato incorporation enriched the phenolic profile and improved antioxidant capacity, with greater retention observed under freeze-drying conditions, particularly in powder-based formulations. Microbiological analysis revealed that freeze-drying preserved higher populations of lactic acid bacteria while suppressing yeast and mold growth. Instrumental aroma analysis demonstrated a clear shift in volatile composition depending on processing conditions, with freeze-drying yielding a more favorable aroma profile compared to sun-drying. Freeze-drying was identified as a superior method for preserving bioactive compounds, microbial viability, and aroma quality in purple sweet potato-enriched tarhana. These findings highlight the functional potential of purple sweet potato as an ingredient in traditional fermented foods and provide a basis for the development of high-quality tarhana formulations.

## 1. Introduction

Purple sweet potato (*Ipomoea batatas* L.) is a distinctive member of the sweet potato family and is rich in various phytochemicals such as phenolic acids, anthocyanins, and carotenoids; it also contains substantial amounts of carbohydrates, minerals, and proteins [1,2]. It has recently attracted considerable attention in functional food research due to its rich bioactive compound content, strong antioxidant activity, and adaptability to diverse climatic conditions [3,4,5]. The addition of anthocyanin and phenolic-rich ingredients such as purple sweet potato has been reported to significantly increase total phenolic content and antioxidant capacity in various food systems, improving color attributes and positively affecting consumer acceptance in cereal-based and fermented food matrices [6,7,8,9]. However, most existing studies have focused on matrices such as bread, extruded products, and fermented beverages, while its application in tarhana—a complex traditional fermented cereal-dairy product—remains largely unexplored [10,11,12].

Tarhana is a traditional fermented cereal-based product widely consumed in Türkiye and surrounding regions. It is typically produced by mixing wheat flour, yogurt, various vegetables (such as tomato and pepper), and spices, followed by spontaneous lactic acid fermentation [13]. During fermentation, lactic acid bacteria (LAB) and yeasts play key roles; LAB lower the pH through acid production, thereby enhancing product safety, while yeasts contribute to the formation of aroma compounds [14,15]. Tarhana is considered a functional food due to its nutritional composition, extended shelf life, and probiotic potential. In recent years, it has gained increasing attention as a potential carrier for functional and health-promoting compounds through the incorporation of plant-based ingredients such as cereals, legumes, fruits, and vegetables [10,11,12].

The drying stage in tarhana production plays a critical role in determining shelf life, microbiological stability, and the retention of heat-sensitive bioactive and aroma compounds. Although traditional sun-drying is economical and widely used, it carries inherent disadvantages including prolonged drying time, risk of environmental contamination, and deterioration of heat-sensitive quality components [16]. Freeze-drying (lyophilization), by contrast, removes water at low temperatures, allowing better preservation of bioactive and aroma compounds [17,18]. Nevertheless, freeze-drying is associated with considerably higher energy consumption and equipment costs, which may limit its widespread adoption in small-scale or traditional food production settings [17]. A rigorous comparative evaluation of both drying methods is therefore necessary to assess their technological performance alongside their practical applicability. While purple sweet potato has been applied as a multifunctional ingredient in dairy-based systems such as Greek yogurt [19], its incorporation into tarhana—in different physical forms and at varying levels—has not been previously reported.

This study was designed to test whether the drying method (sun-drying vs. freeze-drying), incorporation form (puree vs. powder), and the enrichment level (5% and 10%) have different effects on the physicochemical, phenolic, microbiological, and volatile aroma properties of tarhana enriched with purple sweet potatoes, and whether they more effectively preserve the functional quality characteristics of tarhana.

## 2. Materials and Methods

### 2.1. Materials

Purple sweet potatoes (cultivar *İlkmoru*) used in this study were obtained from the Hatay region (Türkiye) in September 2025. Yogurt, cracked wheat and salt used for tarhana production were purchased from local markets in Gaziantep. Analytical grade chemicals and reagents required for the analyses were supplied by nationally operating chemical suppliers.

### 2.2. Methods

#### 2.2.1. Preparation of Purple Sweet Potato Puree

**Figure 1:**
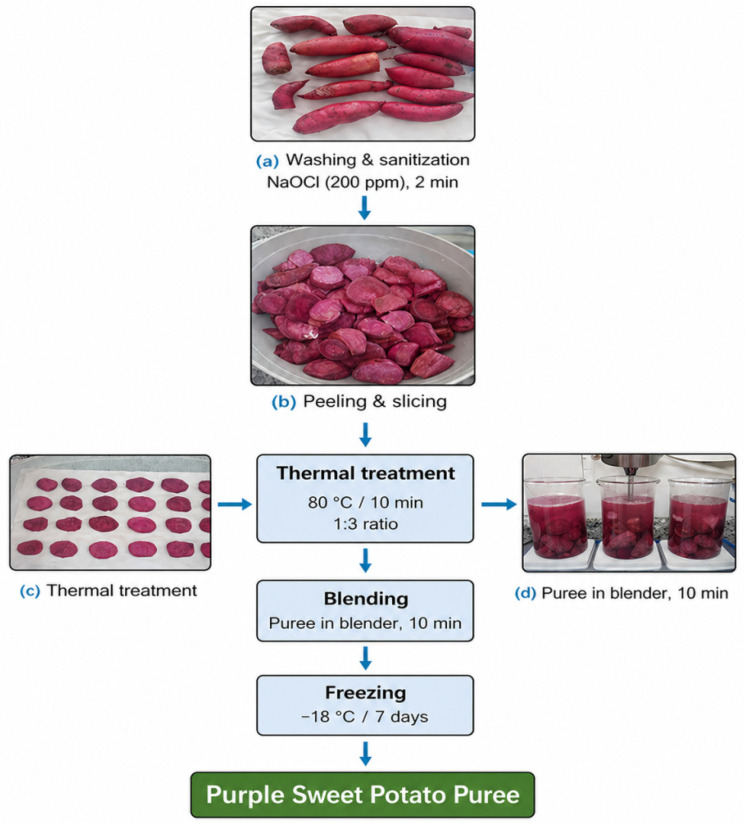
Preparation process of purple sweet potato puree: (**a**) raw purple sweet potatoes after washing and sanitation; (**b**) peeled and sliced samples; (**c**) arrangement prior to thermal treatment; (**d**) thermal processing at 80 °C for 10 min (1:3 *w*/*w*, potato: water ratio).

Purple sweet potatoes were first washed thoroughly and sanitized by immersion in a sodium hypochlorite solution (200 ppm) for 2 min. The samples were then peeled and cut into uniform cross-sectional slices of approximately 3–4 cm in diameter and 0.5–1 cm in thickness by transverse cutting. Thermal treatment was applied by immersing the potato pieces in water at 80 °C for 10 min, using a potato to water ratio of 1:3 (*w*/*w*). The heat treatment was performed to inactivate the endogenous enzymes responsible for enzymatic browning and phenolic degradation in sweet potato tissue—particularly polyphenol oxidase (PPO) and peroxidase [20]—and to reduce the initial microbial load on the surface of purple potatoes, thereby contributing to the microbiological safety of the final product. This temperature-time combination was taken from the method described by Cunha et al. [19]. Following thermal treatment, the cooked potatoes were blended together with the cooking water using a blender (Waring 8011 ES ET1, Waring Commercial, Stamford, CT, USA) for 10 min (Figure 1). The resulting puree was transferred into airtight plastic containers and frozen at −18 °C in a conventional freezer for 7 days.

#### 2.2.2. Preparation of Purple Sweet Potato Powder by Freeze-Drying (Lyophilization)

The purple sweet potato puree was freeze-dried using a laboratory-scale lyophilizer (BK-FD18, BIOBASE, Jinan, Shandong, China). Prior to lyophilization, the puree was spread on stainless-steel trays in a uniform layer of approximately 1 cm thickness and pre-frozen at −70 °C for 24 h. Freeze-drying was then conducted for 120 h at a condenser temperature of −55 °C and a chamber pressure of 5–8 Pa. The resulting lyophilized material was packed in aluminum-laminated polyethylene pouches and stored at 4 °C in the dark until further analysis.

#### 2.2.3. Production of Tarhana Enriched with Purple Sweet Potato

First, the tarhana dough was prepared according to a regional tarhana recipe specific to the Gaziantep region. Dough was prepared by mixing 62.5% strained yogurt, 36.25% cracked wheat, and 1.25% salt. Purple sweet potato puree and freeze-dried powder were incorporated at levels of 5% and 10% on a fresh weight basis relative to the total dough weight. The ingredients were mixed and kneaded until a homogeneous dough was obtained. Purple sweet potatoes were not used in the control samples. The prepared tarhana doughs were fermented in accordance with traditional practices of the Gaziantep region. Fermentation was carried out under ambient conditions of 25 ± 3 °C, reflecting the natural temperature fluctuations characteristic of early autumn ambient conditions in the Gaziantep region. Active temperature control was not applied, in accordance with traditional tarhana production practices. All formulations were fermented simultaneously under the same ambient conditions to ensure uniform exposure across treatment groups [21]. This fixed-duration protocol was applied uniformly across all formulations to ensure standardized and reproducible processing conditions. A 24 h fermentation period at ambient summer temperatures has been reported to yield sufficient acidification and characteristic microbial development in tarhana [13,21]. Following fermentation, tarhana samples were dried using two different drying techniques. For sun-drying (SD), fermented tarhana dough was spread onto clean trays and dried for 5 days at 30–35 °C under natural air circulation. For freeze-drying (FD), fermented samples were first frozen at −70 °C and subsequently dried in a laboratory-scale lyophilizer for 72 h. Following drying, all tarhana samples were stored in sealed airtight containers at 4 °C under dark and dry conditions. All physicochemical, phenolic, antioxidant, microbiological, and volatile aroma analyses were initiated within two weeks of sample production. For each analytical parameter, all sample groups were analyzed within the same analytical session to ensure consistent and comparable measurement conditions across treatments. All analyses were performed in triplicate (*n* = 3) and the results were expressed as mean ± standard deviation.

#### 2.2.4. Analyses Performed on the Produced Tarhana Samples

##### Physicochemical Analyses

Water activity (aw) of tarhana samples was measured at 25 °C using a water activity meter (Rotronic Hygropalm, Bassersdorf, Switzerland). Moisture content was determined using a halogen moisture analyzer (XM 60, Precisa Gravimetrics AG, Dietikon, Switzerland) at 105 °C until constant weight was reached, based on the thermogravimetric loss-on-drying principle in accordance with AOAC Official Method 925.09 [22]. The moisture content and water activity of all dried samples were subsequently determined and are reported in Table 1 (Section 3.1.1). Ash content was determined by incineration at 550 °C for 5 h in a muffle furnace, following AOAC Official Method 923.03 [22]. Protein content was determined by the micro-Kjeldahl method according to AOAC Official Method 960.52 [23]. Nitrogen content was measured and crude protein content was calculated by multiplying the nitrogen value by the conversion factor 5.7, expressed as percentage on a dry matter basis. The pH and titratable acidity of tarhana samples were determined according to Erbaş et al. [13]. For determination pH a 5 g portion of homogenized sample was dispersed in 45 mL of distilled water and the pH was measured with a calibrated digital pH meter (Mettler-Toledo, Greifensee, Switzerland) at room temperature. Titratable acidity was determined by titrating with 0.1 N NaOH and expressed as percent on lactic acid. All analyses were performed in triplicate.

The color properties of tarhana samples were determined using a colorimeter (HunterLab ColorFlex EZ, Hunter Associates Laboratory Inc., Reston, VA, USA) according to the CIELab color system (L*, a*, b*). The instrument was calibrated using standard ceramic tiles prior to measurements. Color measurements were performed at three different points on each sample, and the mean values were recorded.

##### Analysis of Microstructural Produced Tarhana Samples by Scanning Electron Microscopy (SEM)

The microstructure of tarhana samples was examined using a scanning electron microscope (SEM) (ZEISS Gemini SEM 300, Carl Zeiss AG, Oberkochen, Germany). SEM analyses were conducted at the Gaziantep University Ulug Bey High Technology Application and Research Center (ULUTEM) following the methods described by Ranadheera et al. [24]. Prior to imaging, tarhana samples were mounted on carbon adhesive tabs attached to aluminum stubs and coated with a gold/palladium (80:20, *w*/*w*) layer to ensure electrical conductivity. Micrographs were obtained at an accelerating voltage of 20 kV.

##### Analysis of Phenolic Compounds

Individual phenolic compounds were analyzed by HPLC-MS/MS (Agilent Technologies, Inc., Santa Clara, CA, USA) based on the reversed-phase C18 chromatographic framework described by Abad-García et al. [25] and the instrumental parameters reported by Kesen et al. [26], with modifications to the extraction solvent system (methanol:water:formic acid, 70:29:1, *v*/*v*/*v*), mobile phase formic acid concentration (0.1%, *v*/*v*), filter pore size (0.22 µm PTFE membrane), and gradient elution program to adapt the method to the tarhana matrix. A reversed-phase C18 column (250 × 4.6 mm, 5 µm) was used with a flow rate of 0.8 mL/min, column temperature of 30 °C, and injection volume of 10 µL. Detection was carried out by tandem mass spectrometry with electrospray ionization operating in both positive and negative ion modes. Compound identification was performed based on retention times, precursor ions, and characteristic fragment ions by comparison with authentic standards. The analytical method was developed based on established chromatographic principles for reversed-phase C18 separation of phenolic compounds; quantification was performed using external calibration curves with regression coefficients (R^2^) above 0.995 for all compounds, and results were expressed as µg/kg dry matter.

##### Analysis of Antioxidant Activity

Antioxidant activity of tarhana samples was determined using a modified DPPH radical scavenging method as previously described by Xu et al. [27]. A DPPH stock solution was prepared by dissolving 24 mg DPPH in 100 mL methanol and stored at −20 °C until analysis. The working solution was obtained by diluting the stock solution with methanol to achieve an absorbance of 0.70 ± 0.02 at 515 nm. For extraction, 1 g of tarhana sample was mixed with 10 mL aqueous methanol and sonicated for 10 min in an ultrasonic bath. The mixture was then centrifuged at 13,500× *g*, 4 °C for 10 min, and the supernatant was collected. An aliquot of 100 µL of the extract was reacted with 2.9 mL of the DPPH working solution in the dark at room temperature for 60 min to ensure that the DPPH radical scavenging reaction reached a stable endpoint, as recommended for complex food matrices. Absorbance was measured at 515 nm using a UV–Vis spectrophotometer (Shimadzu UV-1800, Shimadzu Corporation, Kyoto, Japan) against methanol as the blank. Antioxidant capacity was quantified using a Trolox calibration curve (5–500 ppm) and expressed as µmol Trolox equivalent per gram of sample (µmol TE/g). All analyses were performed in triplicate.

##### Analysis of Aroma Compounds

*Extraction of Aroma Compounds*

Aroma compounds of tarhana samples were extracted using solid phase microextraction (SPME). A fiber coated with 50/30 µm polydimethylsiloxane/divinylbenzene/carboxen (PDMS/DVB/CAR) was used for extraction. Briefly, 3 g of ground tarhana sample was placed into 10 mL glass vials, and 4-nonanol (internal standard, 10 µL, 10 mg/L in ethanol) was added. The vials were hermetically sealed with PTFE/silicone septa. Samples were equilibrated at 40 °C, and the SPME fiber was exposed to the headspace for 30 min. After extraction, the fiber was immediately desorbed in the GC injector port [28].

*GC-FID and GC-MS Conditions*

Volatile compounds were analyzed using a gas chromatograph (Agilent 6890N, Agilent Technologies, Inc., Santa Clara, CA, USA) equipped with a flame ionization detector (FID) for quantification and coupled to a mass spectrometer (Agilent 5975B VL MSD, Agilent Technologies, Inc., Santa Clara, CA, USA) for identification via a Dean switch splitter system. The column effluent was split equally between the FID and MS detectors. Separation was achieved using a DB-WAX capillary column (60 m × 0.25 mm × 0.25 µm). The injector and detector temperatures were set at 250 °C. The oven temperature program was as follows: initial temperature of 60 °C, increased to 220 °C at 2 °C/min, then to 245 °C at 3 °C/min, and held for 20 min. Helium was used as carrier gas at a constant flow rate of 1.5 mL/min. Injection was performed in splitless mode. The MS operated in electron impact (EI) mode at 70 eV. Ion source and quadrupole temperatures were set at 250 °C and 120 °C, respectively. Mass spectra were recorded in the range of *m*/*z* 29–350. Identification of volatile compounds was carried out by comparing retention indices and mass spectra with those of authentic standards and spectral libraries (Wiley 7.0, NIST-98, and Flavor.2L) [29]. Retention indices were calculated using a homologous n-alkane series (C7–C30) injected under the same chromatographic conditions.

*Quantification of Aroma Compounds*

Quantification of volatile compounds was performed using the internal standard method with 4-nonanol. Calibration curves were constructed using authentic standards, and response factors were calculated. The concentration of each compound was calculated using the following equation:

C_i_ = (A_i_/A_std_) × C_std_ × RF × HF

where C_i_ is the concentration of the aroma compound, A_i_ is the peak area of the compound, A_std_ is the peak area of the internal standard (4-nonanol at a concentration of 41.57 mg/L), C_std_ is the concentration of the internal standard, RF is the response factor, and HF is the conversion factor. All analyses were performed in triplicate.

##### Microbiological Analysis

Tarhana samples (10 g) were homogenized in 90 mL of sterile Ringer’s solution, and serial decimal dilutions were prepared using Ringer’s solution (Merck, Darmstadt, Germany) [30]. Total mesophilic aerobic bacteria (TMAB) were determined by the pour plate technique using Plate Count Agar (Merck), followed by incubation at 30 °C for 3 days [31]. Yeast and mould counts were determined using potato dextrose agar (PDA, Merck) plates following standard plate count methods. The plates were incubated at 25–30 °C for 3–5 days, and colonies were counted after incubation according to internationally recognized microbiological standards [32]. Lactic acid bacteria (LAB), specifically *Lactobacillus* spp., were enumerated using de Man, Rogosa and Sharpe (MRS) agar (Merck) with the pour plate method. Plates were incubated anaerobically at 37 °C for 72 h using an anaerobic jar system with gas-generating sachets to maintain strict anaerobic conditions, in accordance with standard microbiological procedures [33].

#### 2.2.5. Statistical Analysis

All analyses were performed in analytical triplicate (n = 3) on samples obtained from a single production batch per formulation, and results are expressed as mean ± standard deviation. Statistical analyses were carried out using SPSS Statistics software (version 22; IBM Corp., Armonk, NY, USA). The effects of incorporation form (puree vs. powder), incorporation level (5% and 10%), and drying method (sun-drying vs. freeze-drying) on each measured parameter were evaluated separately. Differences among formulation groups within the same drying method were assessed by one-way analysis of variance (ANOVA) followed by Tukey’s Honest Significant Difference (HSD) post hoc test. Differences between drying methods within the same formulation were evaluated by independent samples *t*-test. A significance level of *p* < 0.05 was applied in all tests. Additionally, principal component analysis (PCA) was performed using XLSTAT software (version 2025.2.0; Lumivero, Denver, CO, USA) on both the phenolic compound and volatile aroma compound datasets as a complementary multivariate statistical approach. By simultaneously considering all variables within each dataset, PCA accounts for intercorrelations among parameters and provides an integrated assessment of the main sources of variation among treatment groups, thereby serving as a cross-validation tool for the univariate findings obtained through ANOVA and Tukey’s HSD post hoc test.

## 3. Results and Discussion

### 3.1. Physicochemical Properties

#### 3.1.1. Moisture Content and Water Activity

**Figure 2:**
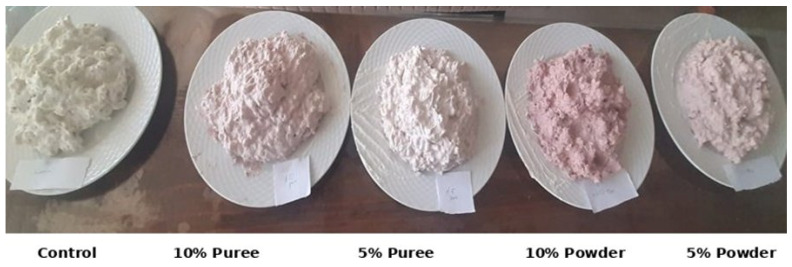
Tarhana doughs enriched with purple sweet potato powder and puree at 5% and 10% incorporation levels, and control.

**Figure 3:**
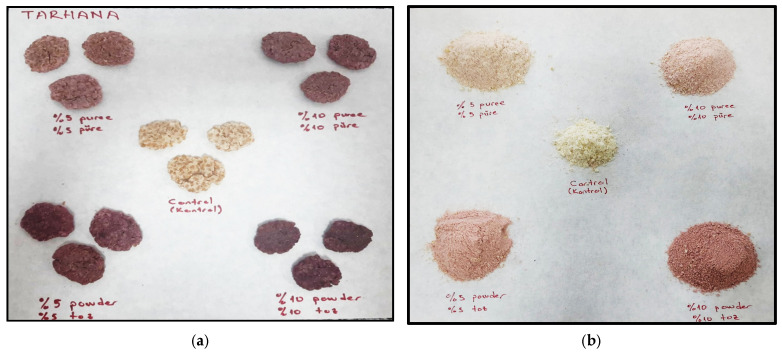
Sun-dried (**a**) and freeze-dried (**b**) tarhana samples.

Table 1 shows the moisture content, water activity (aw), ash, and protein content of tarhana samples enriched with purple sweet potato puree/powder and the control group. Figure 2 shows tarhana dough prepared using control and different ratios (5% and 10%) of purple sweet potato powder and fresh purple sweet potato puree; Figure 3 shows dried tarhana samples obtained by sun-drying and freeze-drying methods.

The moisture content and water activity values of tarhana samples enriched with purple sweet potato varied with drying method and enrichment method. Moisture contents were 5.19–5.31% in sun-dried samples and 3.16–3.51% in freeze-dried samples, while aw values ranged between 0.30–0.40 and 0.12–0.22, respectively; all samples complied with the Turkish Standard moisture limit (<10%) [34]. These findings confirm that freeze-drying is more effective than sun-drying in reducing residual moisture and aw in tarhana, in agreement with previous reports on lyophilized tarhana and similar fermented cereal products [35,36]. A slight increase in moisture content with increasing purple sweet potato level was observed in the sun-dried samples, which may be attributed to the water-binding capacity of plant materials [37]. Compared to traditional products, the water activity levels of tarhana obtained by freeze-drying are lower than the typical range of 0.40–0.60 reported for traditional tarhana [38,39,40]; this may be related to the use of advanced drying technology.

These results demonstrate that the drying method is the most decisive factor influencing the moisture content and water activity of purple sweet potato-enriched tarhana formulations, with all samples complying with the Turkish Standard moisture limit (<10%) [34]. Moisture content was significantly higher in sun-dried samples compared to freeze-dried samples across all formulations and concentrations (*p* < 0.05). In contrast, water activity values were significantly lower in freeze-dried samples, indicating lower water availability, which is associated with reduced microbial growth potential in dried food products.

Ash content ranged from 3.12% to 4.79% in sun-dried samples and from 3.27% to 4.84% in freeze-dried samples. The control samples exhibited ash values between 3.12% and 3.27%, which are in agreement with previously reported ranges for traditional tarhana (3–7%) [40,41]. This consistency confirms that the base formulation (cracked wheat, yogurt, and salt) adequately represents the mineral composition of conventional tarhana.

The incorporation of purple sweet potato significantly increased ash content compared to the control group (*p* < 0.05), indicating an enrichment of total mineral matter. This increase was more pronounced in samples containing powder form than puree. Specifically, the highest ash values were observed in samples with 10% powder addition (4.79% in sun-dried and 4.84% in freeze-dried samples). This trend can be attributed to the higher dry matter content and concentration of minerals in the powdered form, which allows greater incorporation of inorganic constituents into the tarhana matrix. Similar findings have been reported in studies where mineral-rich plant-based ingredients such as wheat germ, bran, and olive leaf powder were added to tarhana formulations, resulting in increased ash content [37,42,43].

In contrast, puree-added samples showed a comparatively moderate increase in ash content. This can be explained by the high moisture content of puree, which dilutes the mineral fraction and reduces the effective incorporation of ash-forming components per unit mass. Nevertheless, even at 10% puree addition, ash values were significantly higher than the control samples, suggesting that purple sweet potato contributes substantially to the mineral profile regardless of its physical form.

From a processing perspective, drying method had a limited but observable influence on ash values. Freeze-dried samples consistently exhibited slightly higher ash contents compared to their sun-dried counterparts within the same formulation. This difference is not due to a chemical alteration of mineral composition but rather to a concentration effect arising from more efficient water removal during freeze-drying. Consequently, the relative proportion of mineral matter in the final dry product increases. Previous studies have similarly reported that drying methods influence measured ash content indirectly through changes in moisture content and matrix structure rather than through direct chemical modification [35,44].

Despite these trends, no statistically significant differences were observed between drying methods or between 5% and 10% concentrations within the same formulation groups (*p* > 0.05). This suggests that while formulation (i.e., the presence and form of purple sweet potato) is the primary determinant of ash content, the effects of concentration level and drying method are secondary within the tested range. The findings demonstrate that purple sweet potato, particularly in powder form, is an effective ingredient for enhancing the mineral content of tarhana. The increase in ash content reflects improved nutritional potential without being adversely affected by the drying technique. These results support the use of purple sweet potato as a functional ingredient in fermented cereal-based foods, contributing to the development of value-added products with enhanced compositional and nutritional properties.

The protein contents of tarhana samples produced by adding purple sweet potato in puree and powder forms and subjected to different drying methods ranged between 12.44% and 13.92% (Table 1). These values are consistent with the protein range (12–18%) reported in the literature for tarhana. It has been widely reported that the protein content of tarhana mainly originates from yogurt and wheat flour and may vary depending on the formulation used [13,45].

In samples enriched with purple sweet potato puree, protein values showed a slight decrease compared to the control sample. This trend was particularly evident in sun-dried samples, where the protein content decreased from 13.09% to 12.44%. This reduction can be explained by the high moisture content of purple sweet potato puree which decreases the total dry matter content of the formulation and results in a relative dilution of proteins derived from yogurt and wheat flour.

In contrast, samples enriched with purple sweet potato powder exhibited protein values similar to or slightly higher than those of the control samples. Notably, the protein content reached 13.92% in freeze-dried samples containing 10% purple sweet potato powder. This can be attributed to the low moisture content of the powder form, which helps maintain the dry matter concentration of the formulation. Likewise, it has been reported that in cereal-based products enriched with vegetable powders, protein percentages tend to remain stable or may slightly increase [13].

Across all samples, freeze-dried tarhana generally exhibited higher protein contents than sun-dried samples. In the control group, protein content was 13.72% in freeze-dried samples and 13.09% in sun-dried samples. This difference can be attributed to the freeze-drying process, which occurs under low temperature and vacuum conditions, thereby minimizing thermal and oxidative damage to proteins [17,46]. In contrast, sun-drying may cause greater alterations in protein structure due to longer drying times and environmental temperature fluctuations. However, despite these numerical differences, no statistically significant differences were observed between drying methods or between different concentrations within the same formulation (*p* > 0.05). Protein analysis results showed slightly higher protein values in powder-enriched samples compared to puree formulations. Previous studies have similarly reported that drying methods may have limited effects on total protein content, but may affect protein functionality and solubility [47].

The pH and titratable acidity of tarhana samples are also presented in Table 1. The pH values ranged from 3.87 to 4.20 in sun-dried samples and from 3.78 to 4.12 in freeze-dried samples, while titratable acidity values ranged from 1.27% to 1.87% and from 1.48% to 2.16% (as lactic acid), respectively. These values are consistent with the ranges reported for traditional tarhana (pH 3.8–4.5; titratable acidity 1.0–2.5%) [13] and confirm that all samples reached a typical post-fermentation acidic state suitable for product stability. The addition of purple sweet potato decreased pH and increased titratable acidity in a concentration-dependent manner, with the lowest pH (3.78–3.87) and highest acidity (1.87–2.16%) observed in 10% powder-enriched samples. Freeze-dried samples consistently showed lower pH and higher titratable acidity compared to sun-dried samples; this is consistent with the better preservation of live LABs during freeze-drying obtained in the present study.

#### 3.1.2. Color Characteristics of Tarhana Samples

Table 2 demonstrates that purple potato addition, formulation type (puree vs. powder), and drying method significantly influenced the color parameters (L*, a*, and b*) of tarhana samples (*p* < 0.05). A consistent decrease in L* values was observed with increasing levels of purple potato incorporation. Control samples exhibited the highest lightness values (SD: 51.97; FD: 88.66), whereas enriched samples showed progressively lower L* values, particularly in powder form. This reduction in lightness is consistent with the known colorimetric behavior of anthocyanin-rich ingredients, which are associated with darker red-purple pigmentation in food matrices [48,49]. Moreover, freeze-dried samples retained higher L* values compared to sun-dried counterparts, indicating better preservation of structural and color integrity due to minimal thermal degradation [17,18].

The a* values increased significantly with purple potato addition, indicating a shift toward red-purple hues. While control samples exhibited low a* values (SD: 4.18; FD: 0.40), enrichment led to pronounced increases, particularly in powder formulations. This observation is consistent with the characteristic red-purple hue associated with the anthocyanin profile of purple sweet potato reported in the literature [50,51], although direct quantification of anthocyanin content in the tarhana samples was not performed in the present study. The more intense color development in powder-enriched samples may reflect a potentially higher effective pigment content per unit mass compared to puree form, owing to the lower moisture content of the powder.

In contrast, b* values decreased with increasing purple potato concentration, reflecting a reduction in yellow tones and a shift toward purple-red coloration. Control samples showed the highest b* values, while enriched samples exhibited progressively lower values. This trend is consistent with the reported masking effect of anthocyanin-rich pigments on yellow pigments such as carotenoids in pigmented plant-based food systems [50,51,52].

Additionally, the drying method played a critical role in color development. Sun-dried samples exhibited lower L* and higher a* values, suggesting more intense color formation, which may be associated with anthocyanin degradation and non-enzymatic browning reactions under prolonged exposure to heat and light [53,54]. In contrast, freeze-drying better preserved the original color characteristics. These variations further highlight the sensitivity of anthocyanins to processing conditions such as temperature, light, and oxygen [55].

According to the results, increasing purple potato concentration, particularly in powder form, enhances redness while reducing lightness and yellowness, leading to a darker and more intensely colored product.

### 3.2. SEM Analysis of Tarhana Samples

**Figure 4:**
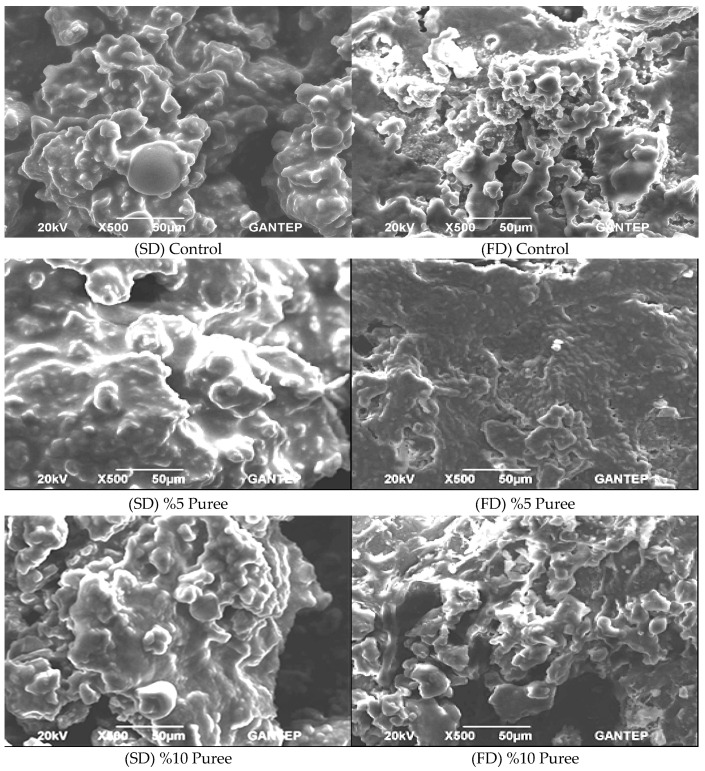

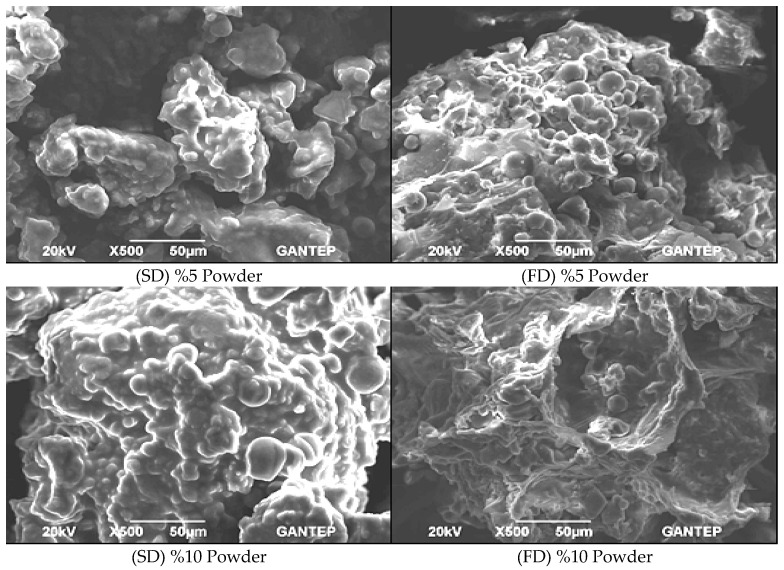
SEM images of freeze-dried and sun-dried tarhana samples enriched with purple sweet potato (puree and powder; 5% and 10%).

Scanning electron microscopy (SEM) images of tarhana samples obtained using freeze-drying and sun-drying methods (control, samples enriched with purple sweet potato in different forms (puree and powder) and at different concentrations (5% and 10%) are presented in Figure 4. All SEM images were obtained at ×500 magnification and revealed distinct morphological differences depending on formulation and processing conditions.

Freeze-dried (FD) tarhana samples exhibited a more open, highly porous, and sponge-like microstructure, whereas sun-dried (SD) samples showed more compact, irregular, and agglomerated surface morphologies. This difference can be explained by the drying mechanism; in freeze-drying, the sublimation of ice crystals preserves void spaces, whereas in sun-drying, the gradual removal of moisture leads to matrix shrinkage and densification. These findings are consistent with studies reporting that controlled low temperature drying preserves porosity, while conventional drying methods result in matrix collapse and particle aggregation [17,36].

The incorporation of purple sweet potato significantly affected the microstructure depending on its form and concentration. When freeze-dried samples were evaluated, the sample containing 5% purple sweet potato puree exhibited a relatively homogeneous structure with limited porosity, whereas the 5% powder-enriched sample showed increased granular structures and pronounced surface heterogeneity. Increasing the purple sweet potato content to 10% resulted in a more expanded and partially thin-walled, highly porous structure in the puree-enriched sample. This observation is consistent with the well-established principle that higher moisture content during freezing may promote the formation of larger ice crystals, resulting in larger voids after sublimation, as reported in previous studies on freeze-dried food matrices [18]. In contrast, the 10% powder-enriched sample exhibited a more heterogeneous microstructure characterized by distinct pores and loosely aggregated structures. These results indicate that the puree form integrates more effectively into the matrix, whereas the powder form promotes a particle-based structure, increasing heterogeneity. Similar findings have been reported for tarhana enriched with plant-based purees such as sour cherry [56].

In sun-dried samples (SD group), the effects of drying conditions were more pronounced. The sample containing 5% purple sweet potato puree exhibited irregular and partially collapsed regions on the surface, whereas the 5% powder-enriched sample showed intense agglomeration and compact particle clusters. This can be explained by the higher moisture content of the puree, which leads to matrix collapse during drying, while the powder acts as a filler within the matrix, promoting particle accumulation [46]. Increasing the purple sweet potato level to 10% resulted in the formation of a thicker and denser matrix in the puree-enriched sample, although porosity remained limited. The 10% powder-enriched sun-dried sample displayed the most irregular and heterogeneous microstructure, characterized by large particle clusters, pronounced surface roughness, and high heterogeneity. These findings are consistent with previous SEM studies on tarhana produced using alternative raw materials, which reported increased agglomeration and heterogeneous particle distribution [57].

The observed agglomeration and surface irregularities are also consistent with the microstructural variations reported in traditional tarhana samples. SEM images of Maraş tarhana have revealed oval amorphous particles and significant surface variability, which were associated with differences in raw material composition, fermentation processes, and drying conditions [58].

Comparative evaluation of drying methods clearly demonstrated that freeze-drying preserves a highly porous and three-dimensional network structure, whereas sun-drying promotes matrix collapse and structural densification. Both the drying method and the form and concentration of purple sweet potato are key factors determining the microstructure of tarhana. Puree incorporation, particularly under freeze-drying conditions, enhances porosity and structural expansion, whereas powder incorporation leads to a more compact and particle-dominated morphology. Increasing incorporation levels further intensifies these effects.

### 3.3. Results of Microbiological Analysis

**Figure 5:**
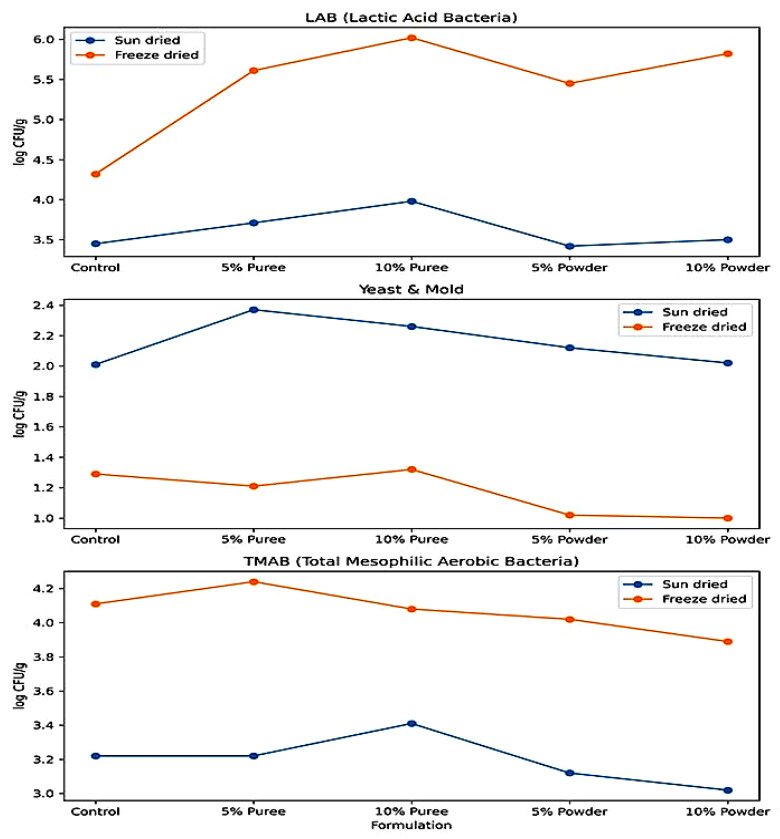
Microbial counts (LAB, yeast–mold, and TMAB) of tarhana samples enriched with purple sweet potato by different drying methods.

The effects of drying method (sun-drying vs. freeze-drying) and the incorporation form of purple sweet potato (powder vs. puree at 5% and 10% levels) on the microbiological quality of tarhana were systematically evaluated. The results demonstrated that both processing parameters significantly influenced the microbial profile, particularly lactic acid bacteria (LAB), total mesophilic aerobic bacteria (TMAB), and yeast–mold (YM) counts (Table 3 and Figure 5).

One of the most prominent findings was the significantly higher LAB counts observed in freeze-dried samples (4.32–6.02 log CFU/g) compared to sun-dried samples (3.45–3.98 log CFU/g). This difference of up to 2 log units in enriched formulations confirms that freeze-drying is highly effective in preserving LAB viability. The protective effect of freeze-drying has been widely attributed to reduced thermal damage and minimized oxidative stress, which help maintain cell membrane integrity and enzymatic activity [59,60]. Similarly, studies on fermented cereal products have reported that LAB survival is highly dependent on post-fermentation processing conditions, particularly drying techniques [13,47].

The incorporation form of purple sweet potato also played a critical role in LAB development. Puree-added samples, especially at 10% concentration, exhibited the highest LAB counts. This observation may be related to the higher water activity and potentially greater nutrient availability in puree-enriched formulations, factors that are known to promote LAB proliferation in fermented food systems. In contrast, powder-added samples showed relatively lower LAB counts, likely due to reduced water activity and limited substrate accessibility [61].

Regarding yeast–mold counts, freeze-dried samples consistently exhibited lower values (1.00–1.32 log CFU/g) compared to sun-dried samples (2.02–2.37 log CFU/g). This reduction can be attributed to the lower water activity and the controlled processing environment of freeze-drying, which inhibits fungal growth. Additionally, powder-added samples showed lower YM counts than puree-added samples, suggesting an enhanced inhibitory effect.

The TMAB results also followed a similar trend, with higher counts in sun-dried samples (3.89–4.24 log CFU/g) compared to freeze-dried samples (3.02–3.42 log CFU/g). This difference is likely due to the open and uncontrolled nature of sun-drying, which increases the risk of environmental contamination and allows prolonged microbial exposure during drying. In contrast, freeze-drying is a closed and controlled process that rapidly reduces moisture content and water activity, thereby limiting microbial proliferation. These findings are consistent with previous studies highlighting that drying conditions significantly influence the overall microbial load in fermented foods [17].

The drying method emerged as the dominant determinant of microbial quality, with freeze-drying preserving beneficial LAB populations while suppressing yeast, mold, and total aerobic bacteria. The form of purple sweet potato played a complementary, dual role: puree promoted LAB growth through higher moisture and nutrient availability, whereas powder enhanced microbial stability via reduced water activity and elevated phenolic concentration. The combination of freeze-drying with powder-based enrichment therefore offers a strategic pathway for producing tarhana with improved microbiological safety and functional quality.

The microstructural differences revealed by SEM provide a mechanistic basis for the divergent water activity and microbial profiles observed. Freeze-drying produced a highly porous, sponge-like matrix through ice sublimation, whereas sun-drying yielded a denser, collapsed structure. The larger internal surface area of freeze-dried matrices distributed residual moisture across many small cavities, lowering water activity to 0.12–0.22, while the sun-dried structure retained water in a more accessible state (a_w_ 0.30–0.40). Although both ranges fall below the critical thresholds for spoilage organisms, the markedly lower a_w_ of freeze-dried samples more effectively suppressed yeast, mold, and total aerobic bacteria. At the same time, the porous freeze-dried matrix appears to have provided a protective microenvironment for LAB, shielding cells from oxidative and dehydration stress and accounting for their higher retained viability (5.45–5.96 log CFU/g).

### 3.4. Phenolic Compounds

The major phenolic acids identified in the tarhana samples were vanillic, syringic, caffeic, *p*-coumaric, ferulic, chlorogenic and fumaric acids (Table 4). The addition of purple sweet potato in either powder or puree form generally increased the concentration of these compounds compared with the control, particularly at the 10% enrichment level.

This finding indicates that the phenolic fraction of the plant ingredient was successfully incorporated into the fermented matrix, which is consistent with earlier studies showing that plant-based enrichment results in higher total phenolic content and phenolic acid derivatives in tarhana and similar cereal-based fermented foods [35,42,62]. The drying method also influenced phenolic retention. Freeze-dried samples contained higher concentrations of most phenolic acids than sun-dried samples, notably caffeic, *p*-coumaric, ferulic and chlorogenic acids. For example, chlorogenic acid increased from 2176–2566 µg/kg in sun-dried tarhana to 3527–4828 µg/kg following freeze-drying. Among the quantified compounds, chlorogenic acid showed the greatest response to drying conditions, which is consistent with its well-documented thermal lability; chlorogenic acid is known to undergo thermal degradation through multiple pathways, yielding numerous degradation products depending on heating time and temperature [63]. Similarly, drying temperature has been reported to significantly influence the retention of phenolic compounds including chlorogenic acid, *p*-coumaric acid, and ferulic acid, with freeze-drying generally achieving superior phenolic retention compared to conventional thermal drying methods including sun-drying [64]. The differential thermal stability of individual phenolic acids may also explain the compound-specific retention patterns observed in the present study: ferulic acid, which possesses a methoxy group on the aromatic ring that confers greater structural stability, showed comparatively better retention under sun-drying conditions than caffeic and chlorogenic acids, which lack this stabilizing substituent and are therefore more susceptible to thermal oxidation. This trend suggests that sublimation-based dehydration may reduce thermal and oxidative degradation of thermolabile phenolics, in agreement with reports that milder or shorter drying processes promote phenolic stability compared with conventional convective drying [65,66].

Powder-enriched samples generally exhibited slightly higher phenolic acid levels than puree-enriched products, likely due to their greater total solids and phenolic density. These results indicate that formulation was the primary determinant of phenolic composition, whereas drying technology modulated the extent of phenolic retention [33].

Beyond quantitative differences in phenolic compound concentrations, the fermentation and drying processes may have induced qualitative biotransformations in the phenolic profile that were not directly assessed in the present study. During lactic acid fermentation, LAB-associated enzymatic activity—including feruloyl esterases and β-glucosidases—has been reported to release bound phenolic acids from plant cell wall matrices, converting esterified forms into free phenolic acids and thereby increasing their extractability and bioavailability in cereal-based fermented foods [67,68]. Furthermore, the elevated temperatures and prolonged drying duration associated with sun-drying may promote oxidative polymerization of phenolic compounds and their participation in Maillard-type reactions with amino acids and reducing sugars, resulting in the formation of bound or complexed phenolic forms with reduced extractability and antioxidant activity [69,70]. These considerations suggest that the observed differences in phenolic compound concentrations between sun-dried and freeze-dried samples may reflect not only differential thermal degradation but also drying-induced changes in phenolic binding status and extractability.

Phenolic compounds exert selective antimicrobial activity [71], which may explain the lower yeast and mold counts observed in powder-enriched, freeze-dried samples, where phenolic concentrations were highest. LAB, by contrast, are generally more tolerant to plant phenolics and can even metabolize hydroxycinnamic acids [72], consistent with the high LAB populations (5.45–5.96 log CFU/g) retained in freeze-dried samples despite their elevated phenolic load. In turn, LAB-associated feruloyl esterases and β-glucosidases release bound phenolic acids from plant cell wall matrices [67], so the higher LAB counts in freeze-dried samples may have further contributed to the greater concentrations of free phenolic acids quantified in these formulations.

### 3.5. Antioxidant Activity

The antioxidant activity of tarhana samples enriched with purple sweet potato and subjected to different drying methods is presented in Table 4. Antioxidant activity values ranged from 9.79 to 10.20 µmol TE/g in sun-dried samples and from 10.14 to 10.68 µmol TE/g in freeze-dried samples. The highest value was observed in the 10% powder freeze-dried group (10.68 µmol TE/g), while the lowest was found in the control sun-dried samples (9.79 µmol TE/g). Statistical analysis revealed that the drying method had a significant effect on antioxidant activity (*p* < 0.05), with freeze-dried samples consistently showing higher values compared to sun-dried counterparts. In contrast, no significant differences were observed among formulation groups (*p* > 0.05), indicating that increasing puree or powder concentration did not significantly alter antioxidant activity within each drying method.

The significantly higher antioxidant activity observed in freeze-dried samples can be attributed to the protective effect of low temperature dehydration, which minimizes the degradation of thermolabile bioactive compounds. Freeze-drying removes water through sublimation under reduced pressure, thereby preserving the structural integrity of antioxidant molecules. In contrast, sun-drying involves prolonged exposure to heat, oxygen, and ultraviolet radiation, which can lead to oxidative degradation of phenolic compounds and a consequent reduction in antioxidant capacity. This finding is consistent with previous studies reporting that conventional drying methods significantly reduce antioxidant activity compared to freeze-drying [17,73].

Although numerically higher antioxidant activity values were observed in purple sweet potato-enriched samples compared to the control, the differences among formulation groups were not statistically significant (*p* > 0.05), indicating that neither the incorporation form nor the concentration level exerted a statistically detectable effect on antioxidant activity within the tested range. This may be explained by the relatively narrow concentration range tested and the potential saturation of the antioxidant capacity of the matrix. Similar findings have been reported in fermented cereal-based products enriched with plant-based bioactive compounds [21]. The drying method, however, emerged as the primary determinant of antioxidant activity, with freeze-dried samples showing significantly higher values compared to sun-dried counterparts (*p* = 0.005), indicating a strong influence of the drying method on antioxidant preservation.

### 3.6. Aroma Profile of Tarhana

Aroma compounds of tarhana samples enriched with purple potato by different drying methods were given in Table 5. The aroma profile of tarhana samples was significantly influenced by both the incorporation form and concentration of purple sweet potato, as well as the applied drying method. Freeze-dried samples exhibited a more balanced and intense aroma profile, whereas sun-dried samples were characterized by higher levels of oxidation-derived compounds.

Aldehydes: Aldehydes, particularly hexanal, were significantly higher in sun-dried samples, indicating enhanced lipid oxidation during drying. Hexanal is widely recognized as a marker of oxidative degradation of unsaturated fatty acids and is associated with green and grassy off-flavors. The elevated levels observed in sun-dried samples can be attributed to prolonged exposure to oxygen, light, and elevated temperatures. Similar trends have been reported in cereal-based and dried food systems [74,75,76]. In contrast, freeze-dried samples showed lower aldehyde levels, suggesting reduced oxidative reactions under low temperature and low pressure conditions. Furthermore, the addition of purple sweet potato, especially in powder form, contributed to a reduction in aldehyde concentrations, likely due to the antioxidant activity of phenolic compounds and anthocyanins, which inhibit lipid oxidation [77].

Alcohols: Alcohols, mainly derived from fermentation processes, showed relatively stable concentrations across different formulations. Compounds such as 3-methyl-1-butanol and 2,3-butanediol are typical metabolites of yeast and lactic acid bacteria and contribute to fermented and malty notes. The limited variation among treatments suggests that purple sweet potato addition did not significantly disrupt fermentation pathways. However, slight decreases in some alcohols in enriched samples may be associated with reduced lipid oxidation or altered microbial metabolism. These findings are consistent with previous studies on fermented cereal products [78].

Esters: Esters, which are responsible for fruity and sweet aroma notes, were generally better preserved in freeze-dried samples. This can be attributed to the mild processing conditions of freeze-drying, which minimize volatilization and degradation losses [17]. The incorporation of purple sweet potato, particularly in powder form, enhanced the levels of certain esters. While the mechanisms underlying this observation were not directly investigated in the present study, differences in matrix structure, water activity, and dry matter content between the two ingredient forms may have contributed to differential aroma compound development and retention during fermentation and drying. Powder-enriched samples, especially at higher concentrations (10%), exhibited more pronounced fruity aroma characteristics.

Organic Acids: Organic acids, particularly acetic acid and hexanoic acid, were generally higher in freeze-dried samples, indicating better retention under low temperature conditions. These compounds are key contributors to sourness and overall flavor balance in fermented foods. The decrease in some acid concentrations with increasing purple sweet potato addition may reflect changes in fermentation dynamics or buffering effects of plant-derived components. Nevertheless, branched-chain acids such as 3-methylbutanoic acid remained at relatively high levels, preserving the characteristic fermented aroma of tarhana.

Terpenes: Limonene, associated with citrus-like aroma, showed a marked increase with purple sweet potato addition, particularly in powder form and at higher concentrations. This suggests that purple sweet potato contributes additional aroma-active compounds to the system. The variability observed in terpene compounds indicates that both matrix composition (puree vs. powder) and drying method influence their stability, consistent with previous findings [79].

Effect of Form (Puree vs. Powder): The form of purple sweet potato significantly affected aroma development. Powder incorporation resulted in higher concentrations of several volatile compounds, particularly esters and terpenes. This can be explained by increased surface area, enhanced mass transfer, and improved release of aroma precursors. In contrast, puree incorporation led to relatively lower aroma intensities. The higher moisture content and more complex matrix structure likely limited the release and diffusion of volatile compounds. Similar effects of matrix structure on aroma release have been reported in semi-solid food systems [80].

Effect of Drying Method (Sun-drying vs. freeze-drying): Drying method emerged as the dominant factor influencing the aroma profile of tarhana. PCA results showed a clear separation between sun-dried and freeze-dried samples along the F1 axis, with freeze-dried samples clustering with esters and organic acids, while sun-dried samples were associated with aldehydes. Freeze-drying preserved thermolabile and aroma-active compounds through low-temperature sublimation under reduced pressure, minimizing oxidation and volatilization losses [17,18]. In contrast, sun-drying promoted lipid oxidation through prolonged exposure to heat, light, and oxygen, resulting in increased formation of aldehydes associated with green, fatty, and off-flavor notes [16].

### 3.7. Results of PCA

**Figure 6:**
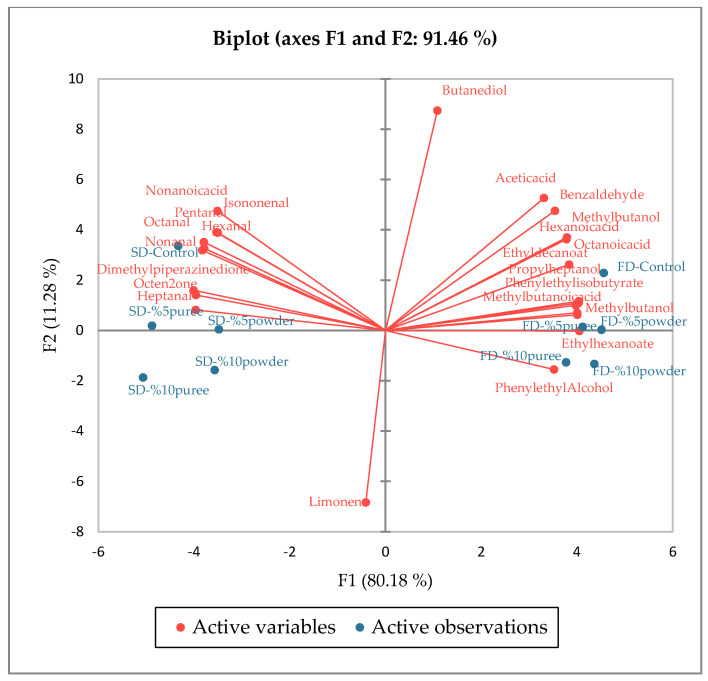
Scores and factor-plane graphs of aroma compounds in tarhana samples.

Principal component analysis (PCA) of the aroma compounds explained 91.46% of the total variance, with F1 and F2 accounting for 80.18% and 11.28%, respectively, indicating a reliable representation of the dataset (Figure 6). A clear separation of samples along the F1 axis was observed based on the drying method. Sun-dried (SD) samples were located on the negative side, while freeze-dried (FD) samples were positioned on the positive side, demonstrating that drying method is the primary factor affecting the volatile profile. In contrast, differences in incorporation form (puree vs. powder) and incorporation level (5% vs. 10%) were limited, as samples within the same drying group clustered closely. FD samples were associated with benzaldehyde, acetic acid, hexanoic and octanoic acids, ethyl decanoate, phenylethyl alcohol, and methylbutanol isomers, which are mainly related to fermentation pathways and contribute to fruity and floral aromas. This indicates that freeze-drying better preserves volatile compounds by minimizing thermal degradation and volatilization losses. In contrast, SD samples were correlated with aldehydes such as hexanal, heptanal, octanal, and nonanal, which are typical products of lipid oxidation. These compounds are associated with green and fatty notes, suggesting that sun-drying promotes oxidative reactions due to exposure to heat, oxygen, and light. The F2 axis contributed to minor variations related to specific compounds (e.g., butanediol and limonene), but did not significantly affect overall sample clustering. The results demonstrate that freeze-drying preserves desirable aroma compounds, whereas sun-drying enhances oxidation-related volatiles, highlighting the critical role of drying method in determining the aroma profile of tarhana enriched with purple sweet potato.

**Figure 7:**
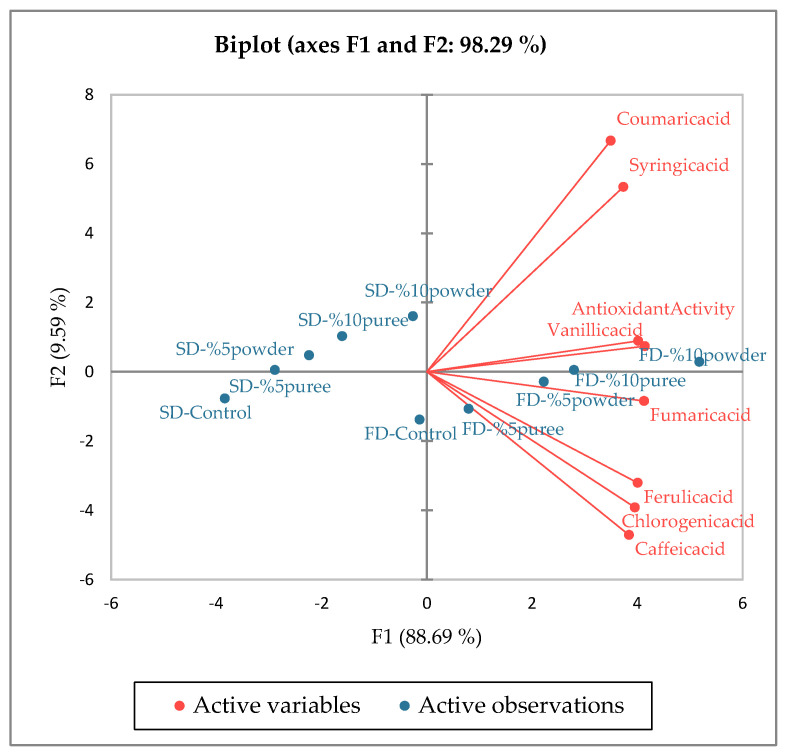
Scores and factor-plane graphs of phenol compounds in tarhana samples.

Principal component analysis (PCA) of phenolic compounds and antioxidant activity explained 98.29% of the total variance, with F1 and F2 accounting for 88.69% and 9.59%, respectively, indicating a highly robust model (Figure 7). A clear separation of samples was observed along the F1 axis according to the drying method. All sun-dried (SD) samples were located on the negative side, whereas freeze-dried (FD) samples were positioned on the positive side, confirming that drying method is the primary factor influencing both phenolic composition and antioxidant activity. Most phenolic compounds, including caffeic acid, chlorogenic acid, ferulic acid, syringic acid, coumaric acid, vanillic acid, and fumaric acid, were strongly associated with FD samples. Notably, antioxidant activity was also positioned in the same direction as these phenolic compounds, indicating a strong positive correlation between total phenolic content and antioxidant capacity. This suggests that freeze-drying effectively preserves bioactive compounds responsible for antioxidant properties by minimizing thermal degradation and oxidative losses. In contrast, SD samples showed weak association with phenolic compounds and antioxidant activity, indicating a reduction in bioactive compounds. This can be attributed to oxidative and thermal degradation during sun-drying, where prolonged exposure to oxygen, light, and higher temperatures promotes the breakdown of phenolic structures. The F2 axis represented minor variations among individual compounds, with coumaric and syringic acids located in the upper region, while caffeic, chlorogenic, and ferulic acids were positioned in the lower region. However, these differences did not significantly affect the overall grouping pattern. Additionally, samples with different addition forms (puree vs. powder) were closely clustered, indicating a limited effect of form, whereas a slight shift in FD samples with higher addition levels (10%) toward the positive F1 direction suggests an increase in phenolic content and antioxidant capacity with increasing enrichment level. Consequently, the PCA results clearly demonstrate that freeze-drying is more effective in preserving both phenolic compounds and antioxidant activity, whereas sun-drying leads to significant losses, highlighting the critical role of processing conditions in maintaining the functional and nutritional quality of purple sweet potato-enriched tarhana.

### 3.8. Practical Implications and Industrial Feasibility

While the present study clearly demonstrates the technical superiority of freeze-drying in preserving phenolic compounds, antioxidant capacity, lactic acid bacteria viability, and aroma quality, the broader applicability of this technology must be evaluated in the context of industrial feasibility and economic viability. Freeze-drying is associated with substantially higher capital investment, and energy consumption has been reported to be substantially higher than that of conventional drying methods [17,18]. These factors considerably limit the adoption of freeze-drying in small-scale, artisanal, or traditional tarhana production settings, where sun-drying remains the economically viable and culturally established practice. From an industrial perspective, the implementation of freeze-drying for tarhana production at commercial scale would require a thorough cost–benefit analysis weighing the enhanced functional quality and potential stability advantages of freeze-dried products against considerably higher production costs. It should also be noted that the quality advantages demonstrated in the present study were obtained under laboratory-scale conditions; scale-up to industrial production may introduce additional variables—including batch size, equipment efficiency, and raw material variability—that could influence final product quality. A viable intermediate approach may involve the use of alternative low-temperature drying technologies, such as vacuum drying or heat pump drying, which can partially replicate the quality-preserving advantages of freeze-drying at considerably lower energy and equipment costs [17,18]. Future techno-economic analyses are therefore recommended to assess the scalability, cost-effectiveness, and commercial viability of low-temperature drying strategies for the production of functional tarhana enriched with bioactive plant-based ingredients.

## 4. Conclusions

This study demonstrated that drying method and incorporation form of purple sweet potato are critical factors influencing the physicochemical, functional, microbiological, and aromatic properties of tarhana. Among these, drying method emerged as the dominant parameter affecting overall product quality. Freeze-drying significantly reduced moisture content and water activity, yielding physicochemical characteristics associated with greater product stability potential. All formulations reached typical post-fermentation pH and titratable acidity values consistent with traditional tarhana standards. It also ensured higher retention of phenolic compounds—particularly chlorogenic, caffeic, and ferulic acids—resulting in enhanced antioxidant capacity. In addition, freeze-drying effectively maintained higher levels of beneficial lactic acid bacteria and suppressed undesirable microorganisms such as yeast and mold, thereby improving microbial quality and safety. Furthermore, aroma analysis revealed that freeze-dried samples retained desirable volatile compounds such as esters, organic acids, and terpenes, whereas sun-dried samples were associated with oxidation-derived aldehydes and potential off-flavors. The incorporation of purple sweet potato successfully enriched tarhana in terms of phenolic composition and antioxidant activity. The form of incorporation played a dual role: puree promoted microbial activity and fermentation dynamics due to its higher moisture, while powder contributed to improved microbial stability and higher retention of bioactive compounds due to its concentrated structure. Increasing incorporation levels generally enhanced functional properties, although their effects were secondary compared to the drying method. Consequently, the combination of freeze-drying and purple sweet potato—particularly in powder form—proved to be the most effective approach for developing a nutritionally enhanced, microbiologically improved, and sensorially superior tarhana. These findings highlight the potential of purple sweet potato as a valuable functional ingredient and provide important technological insights for the optimization of traditional fermented foods. It should be noted that each formulation was produced as a single technological batch, with all measurements performed in analytical triplicate. While this approach is consistent with common practice in exploratory food science research, the absence of independent production replicates represents a limitation of the present study. Future investigations incorporating multiple independent batches are recommended to further confirm the reproducibility of the reported findings. In future studies, sensory evaluations will be conducted to assess consumer acceptance of enriched formulations, and storage stability trials will be performed to monitor the preservation of bioactive compounds and flavor quality over time under defined storage conditions.

## Figures and Tables

**Table 1:** Physical properties of tarhana samples enriched with purple potato by different drying methods.

Physical Properties	Formulation	Conc. (%)	SD	FD
Moisture content (%)	Control	–	5.19 ± 0.01 ^aA^	3.16 ± 0.02 ^dB^
	Puree	5	5.22 ± 0.06 ^aA^	3.45 ± 0.03 ^cB^
		10	5.31 ± 0.03 ^aA^	3.51 ± 0.03 ^cB^
	Powder	5	5.26 ± 0.03 ^aA^	3.25 ± 0.04 ^dB^
		10	5.19 ± 0.02 ^aA^	3.18 ± 0.04 ^dB^
Water activity	Control	–	0.32 ± 0.01 ^bA^	0.12 ± 0.01 ^fB^
	Puree	5	0.34 ± 0.01 ^bA^	0.12 ± 0.01 ^fB^
		10	0.40 ± 0.01 ^aA^	0.15 ± 0.02 ^eB^
	Powder	5	0.39 ± 0.01 ^aA^	0.18 ± 0.01 ^dB^
		10	0.30 ± 0.01 ^cA^	0.22 ± 0.01 ^cB^
Ash content (%)	Control	–	3.12 ± 0.11 ^aA^	3.27 ± 0.12 ^aA^
	Puree	5	4.29 ± 0.28 ^bA^	4.42 ± 0.24 ^bA^
		10	4.44 ± 0.23 ^bcA^	4.58 ± 0.36 ^bA^
	Powder	5	4.54 ± 0.17 ^bcA^	4.69 ± 0.24 ^bA^
		10	4.79 ± 0.19 ^cA^	4.84 ± 0.10 ^bA^
Protein content (%)	Control	–	13.09 ± 0.30 ^bA^	13.72 ± 0.51 ^aA^
	Puree	5	12.99 ± 0.22 ^abA^	13.48 ± 0.53 ^aA^
		10	12.44 ± 0.25 ^aA^	13.37 ± 0.36 ^aA^
	Powder	5	13.21 ± 0.39 ^bA^	13.87 ± 0.36 ^aA^
		10	13.45 ± 0.36 ^bA^	13.92 ± 0.70 ^aA^
pH	Control	–	4.20 ± 0.01 ^dA^	4.12 ± 0.02 ^dB^
	Puree	5	4.07 ± 0.01 ^cA^	3.97 ± 0.03 ^cB^
		10	3.93 ± 0.03 ^bA^	3.88 ± 0.02 ^bB^
	Powder	5	3.95 ± 0.01 ^bA^	3.85 ± 0.01 ^bA^
		10	3.87 ± 0.01 ^aA^	3.78 ± 0.01 ^aB^
Titratable acidity (%)	Control	–	1.27 ± 0.05 ^aA^	1.48 ± 0.04 ^aB^
	Puree	5	1.48 ± 0.04 ^bA^	1.69 ± 0.03 ^bB^
		10	1.69 ± 0.06 ^cA^	1.92 ± 0.03 ^cB^
	Powder	5	1.60 ± 0.03 ^cA^	1.81 ± 0.03 ^cB^
		10	1.87 ± 0.03 ^dA^	2.16 ± 0.04 ^eB^

Values are presented as mean ± standard deviation (n = 3). Means followed by different lowercase letters (a–f) within the same column are significantly different among treatments (*p* < 0.05), while different uppercase letters (A, B) in the same row indicate significant differences between drying methods within the same formulation (*p* < 0.05).

**Table 2:** Color characteristics of purple potato-enriched tarhana by drying method.

Color Value	Formulation	Conc. (%)	SD	FD
L*	Control	–	51.97 ± 0.20 ^eA^	88.66 ± 0.09 ^eB^
	Puree	5	48.18 ± 0.13 ^dA^	81.70 ± 0.15 ^dB^
		10	41.91 ± 0.04 ^cA^	79.53 ± 0.19 ^cB^
	Powder	5	37.49 ± 1.08 ^bA^	73.79 ± 0.03 ^bB^
		10	31.80 ± 2.02 ^aA^	69.24 ± 1.96 ^aB^
a*	Control	–	4.18 ± 0.04 ^aA^	0.40 ± 0.03 ^aB^
	Puree	5	10.17 ± 0.07 ^bA^	5.24 ± 0.01 ^bB^
		10	11.26 ± 0.03 ^cA^	6.68 ± 0.06 ^cB^
	Powder	5	14.37 ± 0.20 ^eA^	9.87 ± 0.03 ^dB^
		10	13.52 ± 0.28 ^dA^	11.63 ± 0.08 ^eB^
b*	Control	–	18.03 ± 0.02 ^dA^	13.32 ± 0.05 ^dB^
	Puree	5	12.81 ± 0.26 ^bA^	11.71 ± 0.08 ^cB^
		10	12.53 ± 0.07 ^aA^	11.30 ± 0.14 ^bB^
	Powder	5	11.14 ± 1.02 ^aA^	11.27 ± 0.17 ^bB^
		10	10.73 ± 1.03 ^aA^	10.54 ± 0.04 ^aB^

Values are presented as mean ± standard deviation (n = 3). Means followed by different lowercase letters (a–e) within the same column are significantly different among treatments (*p* < 0.05), while different uppercase letters (A, B) in the same row indicate significant differences between drying methods within the same formulation (*p* < 0.05).

**Table 3:** Microbiological properties of tarhana samples enriched with purple potato by different drying methods.

Microbial Properties	Formulation	Conc. (%)	SD	FD
LAB (log CFU/g)	Control	–	3.45 ± 0.09 ^aA^	4.32 ± 0.13 ^aB^
	Puree	5	3.71 ± 0.05 ^aA^	5.61 ± 0.25 ^bcB^
		10	3.98 ± 0.27 ^aA^	6.02 ± 0.16 ^cB^
	Powder	5	3.42 ± 0.21 ^aA^	5.45 ± 0.13 ^bB^
		10	3.50 ± 0.28 ^aA^	5.82 ± 0.15 ^bcB^
Yeast & Mold (log CFU/g)	Control	–	2.01 ± 0.02 ^aA^	1.29 ± 0.16 ^bA^
	Puree	5	2.37 ± 0.17 ^aA^	1.21 ± 0.11 ^abB^
		10	2.26 ± 0.18 ^aA^	1.32 ± 0.15 ^bB^
	Powder	5	2.12 ± 0.20 ^aA^	1.02 ± 0.06 ^aB^
		10	2.02 ± 0.19 ^aA^	1.00 ± 0.07 ^aB^
TMAB (log CFU/g)	Control	–	4.11 ± 0.17 ^aA^	3.22 ± 0.24 ^aB^
	Puree	5	4.24 ± 0.18 ^aA^	3.22 ± 0.24 ^aB^
		10	4.08 ± 0.15 ^aA^	3.41 ± 0.19 ^aB^
	Powder	5	4.02 ± 1.32 ^aA^	3.12 ± 0.09 ^aB^
		10	3.89 ± 0.19 ^aA^	3.02 ± 0.16 ^aB^

Values are presented as mean ± standard deviation (n = 3). Different lowercase letters (a–c) within the same column indicate significant differences among formulations, different uppercase letters (A, B) indicate significant differences between drying methods (*p* < 0.05).

**Table 4:** Phenolic compounds (µg/kg) and antioxidant activity (µmolTE/g) of tarhana samples enriched with purple potato by different drying methods.

Compound	Formulation	Conc. (%)	SD	FD	Compound	Formulation	Conc. (%)	SD	FD
Vanillic acid	Control	–	1634.31 ± 31.50 ^aA^	1712.92 ± 20.90 ^aB^	Syringic acid	Control	–	486.41 ± 23.03 ^aA^	506.27 ± 11.36 ^aB^
	Puree	5	1665.51 ± 21.82 ^abA^	1738.26 ± 31.04 ^abB^		Puree	5	502.05 ± 10.60 ^bA^	522.21 ± 25.03 ^abA^
		10	1697.22 ± 17.54 ^bcA^	1760.00 ± 22.27 ^bB^			10	518.04 ± 11.70 ^abA^	545.74 ± 17.17 ^bcA^
	Powder	5	1680.27 ± 20.83 ^bcA^	1764.37 ± 20.99 ^bB^		Powder	5	514.83 ± 25.71 ^abA^	542.53 ± 9.54 ^bcA^
		10	1715.29 ± 21.72 ^cA^	1886.90 ± 18.06 ^cB^			10	545.05 ± 6.00 ^bA^	565.99 ± 10.38 ^cB^
Caffeic acid	Control	–	1730.73 ± 23.20 ^aA^	2152.56 ± 18.33 ^aB^	*p*-Coumaric acid	Control	–	904.00 ± 18.84 ^aA^	997.97 ± 10.44 ^aB^
	Puree	5	1745.86 ± 24.22 ^abA^	2193.70 ± 65.50 ^abB^		Puree	5	987.45 ± 12.08 ^bA^	1024.78 ± 22.85 ^aA^
		10	1786.52 ± 19.83 ^bcA^	2278.24 ± 23.3 ^cB^			10	1096.32 ± 10.82 ^dA^	1166.90 ± 16.99 ^cB^
	Powder	5	1777.82 ± 37.33 ^bcA^	2252.62 ± 19.58 ^bcB^		Powder	5	1021.59 ± 21.65 ^cA^	1112.25 ± 13.31 ^bB^
		10	1807.85 ± 24.16 ^cA^	2376.21 ± 25.92 ^dB^			10	1135.56 ± 15.51 ^eA^	1214.17 ± 13.16 ^dB^
Ferulic acid	Control	–	1643.01 ± 15.32 ^aA^	1965.05 ± 19.19 ^aB^	Chlorogenic acid	Control	–	2176.16 ± 35.05 ^aA^	3526.65 ± 30.22 ^aB^
	Puree	5	1685.83 ± 20.2 ^abA^	1998.69 ± 22.62 ^aB^		Puree	5	2191.56 ± 20.84 ^aA^	3765.38 ± 29.98 ^bB^
		10	1721.74 ± 35.78 ^bA^	2068.60 ± 24.20 ^bB^			10	2221.47 ± 32.53 ^aA^	3994.08 ± 45.72 ^dB^
	Powder	5	1702.46 ± 26.78 ^bA^	2067.49 ± 29.82 ^bB^		Powder	5	2208.62 ± 36.01 ^aA^	3928.95 ± 34.47 ^cB^
		10	1798.82 ± 23.17 ^cA^	2176.73 ± 11.57 ^cB^			10	2565.43 ± 51.26 ^bA^	4827.59 ± 25.74 ^eB^
Fumaric acid	Control	–	1888.21 ± 10.99 ^aA^	2018.27 ± 26.77 ^aB^					
	Puree	5	1907.65 ± 30.73 ^abA^	2065.02 ± 24.76 ^abB^					
		10	1948.15 ± 29.03 ^bcA^	2168.46 ± 25.63 ^cB^					
	Powder	5	1932.88 ± 19.05 ^abA^	2112.40 ± 35.92 ^bB^					
		10	1989.85 ± 22.41 ^cA^	2249.70 ± 21.90 ^dB^					
Antioxidant activity (µmolTE/g)	Control	–	9.79 ± 0.23 ^aA^	10.14 ± 0.24 ^aB^					
	Puree	5	9.85 ± 0.29 ^aA^	10.21 ± 0.35 ^aB^					
		10	10.01 ± 0.40 ^aA^	10.47 ± 0.23 ^aB^					
	Powder	5	9.92 ± 0.43 ^aA^	10.39 ± 0.22 ^aB^					
		10	10.20 ± 0.02 ^aA^	10.68 ± 0.26 ^aB^					

Values are presented as mean ± standard deviation (n = 3). Different lowercase letters (a–e) within each column denote significant differences among formulations, whereas different uppercase letters (A, B) within each row indicate significant differences between drying methods (*p* < 0.05).

**Table 5:** Aroma compounds of tarhana samples enriched with purple potato by different drying methods.

Compound	Formulation	Conc. (%)	SD	FD	Compound	Formulation	Conc. (%)	SD	FD
Hexanal	Control	–	1259.84 ± 39.88 ^cA^	996.38 ± 14.92 ^dB^	Heptanal	Control	–	18.21 ± 0.61 ^bA^	14.62 ± 0.31 ^cB^
	Puree	5	1202.52 ± 11.00 ^bA^	925.11 ± 20.32 ^cB^		Puree	5	18.02 ± 0.54 ^abA^	14.25 ± 0.70 ^bcB^
		10	1195.43 ± 14.04 ^bA^	882.56 ± 30.85 ^bB^			10	18.56 ± 0.68 ^bA^	14.84 ± 0.40 ^cB^
	Powder	5	1147.22 ± 29.28 ^aA^	826.52 ± 20.79 ^aB^		Powder	5	17.86 ± 0.62 ^aA^	13.42 ± 0.45 ^abB^
		10	1104.23 ± 17.37 ^aA^	802.63 ± 15.22 ^aB^			10	17.02 ± 0.43 ^aA^	13.07 ± 0.28 ^aB^
Pentanol	Control	–	53.61 ± 3.10 ^bA^	45.57 ± 2.79 ^dB^	Limonene	Control	–	245.73 ± 5.16 ^aA^	356.21 ± 10.95 ^bB^
	Puree	5	52.52 ± 1.51 ^bA^	42.42 ± 1.46 ^cdA^		Puree	5	326.23 ± 11.69 ^bA^	334.23 ± 21.88 ^abB^
		10	50.78 ± 2.44 ^abA^	39.96 ± 1.95 ^cB^			10	381.28 ± 11.84 ^cA^	315.42 ± 13.72 ^aB^
	Powder	5	50.63 ± 2.32 ^aA^	36.16 ± 0.64 ^bB^		Powder	5	410.04 ± 9.90 ^dA^	351.35 ± 11.88 ^bB^
		10	47.55 ± 1.39 ^aA^	32.43 ± 1.10 ^aB^			10	438.54 ± 16.50 ^dA^	363.24 ± 16.71 ^bB^
Ethyl hexanoate	Control	–	10.25 ± 0.03 ^bA^	36.43 ± 0.71 ^aB^	3-Methyl butanol	Control	–	27.54 ± 0.69 ^aA^	47.83 ± 1.94 ^bB^
	Puree	5	9.12 ± 0.45 ^aA^	36.02 ± 1.47 ^aB^		Puree	5	26.62 ± 1.30 ^aA^	44.38 ± 2.29 ^abB^
		10	8.96 ± 0.43 ^aA^	35.86 ± 2.71 ^aB^			10	26.06 ± 1.09 ^aA^	40.43 ± 2.12 ^aB^
	Powder	5	10.02 ± 0.48 ^bA^	36.25 ± 2.04 ^aB^		Powder	5	27.48 ± 1.41 ^aA^	47.17 ± 1.04 ^bB^
		10	9.86 ± 0.30 ^bA^	36.53 ± 1.85 ^aB^			10	27.36 ± 1.82 ^aA^	47.06 ± 3.46 ^bB^
Octanal	Control	–	210.76 ± 9.37 ^dA^	116.42 ± 6.24 ^cB^	Nonanal	Control	–	564.89 ± 19.88 ^cA^	417.93 ± 18.84 ^bB^
	Puree	5	188.10 ± 8.98 ^cA^	103.88 ± 3.90 ^abB^		Puree	5	538.62 ± 15.79 ^bcA^	396.88 ± 13.94 ^abB^
		10	172.55 ± 9.27 ^bA^	98.32 ± 6.83 ^aB^			10	512.88 ± 10.09 ^abA^	373.44 ± 17.46 ^aB^
	Powder	5	164.89 ± 8.10 ^aA^	112.23 ± 8.45 ^bcB^		Powder	5	505.57 ± 17.29 ^aA^	410.06 ± 12.15 ^bB^
		10	155.47 ± 7.09 ^aA^	107.44 ± 6.42 ^bcB^			10	486.12 ± 13.48 ^aA^	406.26 ± 13.58 ^bB^
(*E*)-3-Octen-2-one	Control	–	53.78 ± 5.06 ^cA^	–	2-Propyl-1-heptanol	Control	–	375.26 ± 5.25 ^cA^	523.01 ± 11.93 ^bB^
	Puree	5	50.14 ± 3.03 ^bcA^	–		Puree	5	355.72 ± 13.13 ^bA^	501.77 ± 11.41 ^abB^
		10	44.63 ± 1.99 ^bA^	–			10	332.82 ± 11.20 ^aA^	486.99 ± 16.80 ^aB^
	Powder	5	38.45 ± 1.02 ^aA^	–		Powder	5	366.01 ± 11.60 ^bcA^	518.93 ± 13.11 ^bB^
		10	34.59 ± 3.39 ^aA^	–			10	360.47 ± 2.49 ^bcA^	513.40 ± 11.28 ^bB^
Acetic acid	Control	–	198.71 ± 3.83 ^cA^	246.89 ± 9.60 ^cB^	Benzaldehyde	Control	–	193.61 ± 6.46 ^cA^	223.16 ± 12.27 ^bB^
	Puree	5	175.16 ± 9.40 ^bA^	226.84 ± 7.41 ^bB^		Puree	5	168.09 ± 8.39 ^bA^	207.09 ± 9.95 ^abB^
		10	161.25 ± 10.66 ^aA^	215.32 ± 12.30 ^abB^			10	153.43 ± 6.22 ^aA^	200.72 ± 10.49 ^aB^
	Powder	5	185.84 ± 9.91 ^bA^	208.61 ± 10.73 ^abB^		Powder	5	188.95 ± 9.27 ^bA^	213.15 ± 14.62 ^abB^
		10	179.38 ± 3.81 ^bA^	197.04 ± 9.67 ^aB^			10	171.01 ± 6.44 ^bA^	206.86 ± 7.87 ^abB^
Isononenal	Control	–	129.39 ± 8.06 ^cA^	101.07 ± 5.38 ^dB^	2,3-Butanediol	Control	–	569.83 ± 15.89 ^aA^	575.05 ± 9.35 ^aA^
	Puree	5	123.76 ± 4.96 ^bcA^	91.05 ± 2.57 ^cB^		Puree	5	550.27 ± 12.53 ^aA^	556.08 ± 15.47 ^aA^
		10	114.18 ± 5.40 ^abA^	82.67 ± 3.40 ^bB^			10	541.18 ± 15.03 ^aA^	543.35 ± 25.24 ^aA^
	Powder	5	117.66 ± 6.19 ^aA^	94.32 ± 4.38 ^cdB^		Powder	5	562.02 ± 13.07 ^aA^	564.09 ± 16.14 ^aA^
		10	108.77 ± 4.93 ^aA^	71.45 ± 3.01 ^aB^			10	553.52 ± 15.60 ^aA^	558.51 ± 18.25 ^aA^
3-Methyl-2-butanol	Control	–	311.28 ± 12.73 ^cA^	365.42 ± 9.09 ^bB^	Ethyl decanoate	Control	–	108.85 ± 5.2 ^bA^	258.14 ± 10.10 ^bB^
	Puree	5	282.79 ± 9.42 ^abA^	348.32 ± 13.08 ^abB^		Puree	5	92.08 ± 3.35 ^aA^	232.36 ± 15.01 ^abB^
		10	264.70 ± 9.10 ^aA^	334.37 ± 11.66 ^aB^			10	85.14 ± 5.36 ^aA^	220.76 ± 16.75 ^aB^
	Powder	5	304.45 ± 11.85 ^bA^	357.05 ± 17.34 ^abB^		Powder	5	102.47 ± 6.95 ^aA^	243.82 ± 13.03 ^abB^
		10	285.26 ± 10.41 ^bA^	342.63 ± 19.95 ^abB^			10	94.08 ± 4.28 ^aA^	235.14 ± 15.86 ^abB^
3,6-Dimethylpiperazine-2,5-dione	Control	–	42.16 ± 2.12 ^bA^	20.19 ± 0.92 ^bB^	3-Methyl butanoic acid	Control	–	256.26 ± 6.17 ^dA^	617.14 ± 10.29 ^aB^
	Puree	5	38.13 ± 1.06 ^aA^	19.13 ± 0.57 ^abB^		Puree	5	221.12 ± 10.41 ^cA^	618.99 ± 19.08 ^aB^
		10	36.95 ± 1.87 ^aA^	18.44 ± 0.50 ^aB^			10	205.23 ± 9.92 ^bA^	608.75 ± 11.09 ^aB^
	Powder	5	41.10 ± 3.14 ^aA^	20.01 ± 1.03 ^bB^		Powder	5	196.23 ± 6.19 ^aA^	611.20 ± 20.36 ^aB^
		10	38.23 ± 1.77 ^aA^	19.82 ± 0.28 ^bB^			10	186.56 ± 7.32 ^aA^	594.14 ± 19.64 ^aB^
Hexanoic acid	Control	–	305.25 ± 10.80 ^cA^	418.46 ± 15.89 ^cB^	2-Phenylethyl isobutyrate	Control	–	206.58 ± 10.74 ^bA^	435.33 ± 15.23 ^cB^
	Puree	5	264.13 ± 16.91 ^bA^	365.67 ± 14.02 ^aB^		Puree	5	203.16 ± 13.22 ^bA^	439.09 ± 20.80 ^cB^
		10	232.89 ± 9.57 ^aA^	341.68 ± 16.95 ^aB^			10	186.58 ± 7.10 ^aA^	412.07 ± 20.65 ^bcB^
	Powder	5	281.48 ± 7.12 ^bA^	394.48 ± 15.70 ^bcB^		Powder	5	201.41 ± 8.36 ^aA^	401.80 ± 12.48 ^abB^
		10	273.54 ± 10.82 ^bA^	368.38 ± 10.84 ^abB^			10	170.24 ± 4.98 ^aA^	378.95 ± 9.62 ^aB^
Phenylethyl alcohol	Control	–	259.10 ± 13.40 ^abA^	327.21 ± 13.78 ^aB^	Octanoic acid	Control	–	277.40 ± 13.09 ^cA^	450.72 ± 17.36 ^cB^
	Puree	5	271.92 ± 9.30 ^bA^	343.00 ± 19.55 ^abB^		Puree	5	241.65 ± 9.67 ^aA^	425.94 ± 11.98 ^cB^
		10	298.52 ± 8.34 ^cA^	359.19 ± 14.97 ^bB^			10	224.40 ± 8.09 ^aA^	389.72 ± 17.83 ^bB^
	Powder	5	262.22 ± 12.35 ^aA^	338.91 ± 14.42 ^abB^		Powder	5	268.95 ± 8.86 ^bA^	374.61 ± 13.55 ^abB^
		10	248.35 ± 9.48 ^aA^	316.11 ± 12.71 ^aB^			10	251.47 ± 10.50 ^bA^	356.27 ± 11.20 ^aB^
Nonanoic acid	Control	–	277.40 ± 13.15 ^cA^	179.25 ± 9.69 ^bB^					
	Puree	5	262.78 ± 7.63 ^bcA^	173.46 ± 10.32 ^bB^					
		10	245.86 ± 10.38 ^bA^	167.12 ± 8.96 ^bB^					
	Powder	5	202.85 ± 5.13 ^aA^	150.89 ± 4.42 ^aB^					
		10	194.99 ± 6.46 ^aA^	144.46 ± 5.80 ^aB^					

Values are presented as mean ± standard deviation (n = 3). Different lowercase letters (a–d) within each column denote significant differences among formulations, whereas different uppercase letters (A, B) within each row indicate significant differences between drying methods (*p* < 0.05).

## Data Availability

The original contributions presented in the study are included in the article, further inquiries can be directed to the corresponding authors.
